# Irisin protects cardiomyocytes against hypoxia/reoxygenation injury via attenuating AMPK mediated endoplasmic reticulum stress

**DOI:** 10.1038/s41598-022-11343-0

**Published:** 2022-05-06

**Authors:** Rongchuan Yue, Mingming Lv, Meide Lan, Zaiyong Zheng, Xin Tan, Xuemei Zhao, Yulong Zhang, Jun Pu, Lei Xu, Houxiang Hu

**Affiliations:** 1 Department of Cardiology, Affiliated Hospital of North Sichuan Medical College, No. 63, Wenhua Road, Shunqing District, Nanchong, 637000 Sichuan People’s Republic of China; 2 Academician Workstation, Affiliated Hospital of North Sichuan Medical College, Nanchong, 637000 Sichuan People’s Republic of China; 3 Cardiovascular Research Center, Affiliated Hospital of North Sichuan Medical College, Nanchong, 637000 People’s Republic of China; 4 Department of Cardiology, Central Hospital of Guangyuan, No. 16, Jing Alley, Lizhou District, Guangyuan, 628000 Sichuan People’s Republic of China; 5 Anesthesiology Department, Affiliated Hospital of North Sichuan Medical College, Nanchong, 637000 People’s Republic of China

**Keywords:** Cell biology, Cardiology

## Abstract

Endoplasmic reticulum (ER) stress plays a central role in myocardial ischemia/reperfusion (I/R) injury. Irisin has been reported to have protective properties in ischemia disease. In this study, we aimed at investigating whether irisin could alleviate myocardial I/R injury by ER stress attenuation. The in vitro model of hypoxia/reoxygenation (H/R) was established, which resembles I/R in vivo. Cell viability and apoptosis were estimated. Expressions of cleaved caspase-3, cytochrome c, GRP78, pAMPK, CHOP, and eIF2α were assessed by western blot. Our results revealed that pre-treatment with irisin significantly decreased cytochrome c release from mitochondria and caspase-3 activation caused by H/R. Irsin also reduced apoptosis and increased cell viability. These effects were abolished by AMPK inhibitor compound C pre-treatment. Also, GRP78 and CHOP expressions were up-regulated in the H/R group compared to the control group; however, irisin attenuated their expression. The pAMPK level was significantly decreased compared to the control, and this effect could be partly reversed by metformin pre-treatment. These results suggest that ER stress is associated with cell viability decreasing and cardiomyocytes apoptosis induced by H/R. Irisin could efficiently protect cardiomyocytes from H/R-injury via attenuating ER stress and ER stress-induced apoptosis.

## Introduction

Cardiovascular disease is one of the most prevalent diseases in developed and developing countries, with high morbidity and mortality rates^1^. It has been demonstrated that cardiomyocyte apoptosis is involved in cardiac dysfunction and chronic heart diseases^2,3^, such as heart failure, ischemia/reperfusion injury, myocardial infarction, and dilated cardiomyopathy.

Ischemia/reperfusion-injury induced by coronary revascularization therapy has been a significant focus of medical research because of its long-term impact on cardiac dysfunction^4,5^. There is growing evidence that mitochondrial dysfunction plays an essential role in cell injury induced by myocardial ischemia/reperfusion and reactive oxygen species. The release of apoptosis-inducing factor (AIF), cytochrome c, and the decrease of membrane ΔΨ are all mechanisms that lead to mitochondrial dysfunction and apoptotic cell death^6–8^. AMP-activated protein kinase (AMPK) is activated to protect cardiomyocytes from I/R injury via regulating mitochondrial function and endoplasmic reticulum homeostasis^9,10^.

Previous studies reported that endoplasmic reticulum (ER) stress-associated apoptosis is involved in I/R injury. Therefore, it is possible to alleviate I/R injury via attenuating ER stress^11,12^. For example, antioxidants, such as pineal secreted hormone melatonin and inartificial pigment lycopene can protect primary cultured neonatal cardiomyocytes against hypoxia/reoxygenation (H/R) injury by attenuating ER stress and maintaining mitochondrial functions^6,13,14^. In addition, irisin has also been shown to reduce oxidative stresses and apoptosis in different models^15,16^.

Irisin, a type I membrane protein encoded by the FNDC5 (fibronectin domain-containing 5 protein) gene, participates in mitochondrial biogenesis and oxidative metabolism^15,17^. Recent evidence has indicated that irisin could induce white adipose tissue's browning, which could be used as a therapeutic tool for metabolic disorders^18,19^. Moreover, the systemic administration of irisin ameliorated atherosclerosis in an apoE (−/−) diabetic mouse model and protected against endothelial injury, indicating that irisin could benefit atherosclerotic vascular diseases^20^.

While irisin's lipogenic effect may correlate with its chronic actions^17,21–23^, no study tests its acute effect on the heart. Therefore, we tested the hypothesis that irisin functions as a myokine to alleviate I/R injury by attenuating ER stress and ER stress-associated apoptosis.

## Materials and methods

### Cell culture

Primary cultures of ventricular cardiomyocytes were prepared from neonatal C57BL/6 mice (Animal Center of the North Sichuan Medical College, Nanchong, China) according to the method in our previous study with few modifications^6^. After being disinfected with 70% ethanol, the neonatal mice were euthanized by decapitation; and the heart was quickly removed and placed in an ice-cold PBS buffer. The heart was washed with ice-cold PBS buffer 3 times and then cut into small pieces in dissociation buffer (containing 0.1% trypsin and 99.9% PBS). After, it was digested for 8 min. Next, the supernatant was transferred to a centrifuge tube, mixed with 2 ml fetal bovine serum, and preserved at 4 °C. The remaining tissue was digested in a solution containing 0.08% collagenase and 99.92% M199 medium (HyClone SH30253.01B). After digestion at 37 °C for 30 min, the cell suspension was mixed with the supernatants, filtrated with a 200X screen, and centrifuged at 300×*g* for 5 min. Next, after washing the precipitate with PBS buffer twice, the dissociated cells were replated in a culture flask at 37 °C for 1 h to enrich the culture's cardiomyocytes. The cells were collected in a medium consisting of M199 supplemented with 50 units/ml penicillin/streptomycin, 10% fetal bovine serum, and 0.1 mM Bromodeoxyuridine (BrdU). After 24 h, replace the BrdU-free DMEM medium. BrdU is commonly used to block the mitosis of non-myocyte cells in primary cardiomyocyte culture. Previous research indicated that BrdU could reduce glycogen which means it may have an impact on AMPK^24^.To made its influence less obvious, the control groups were settled set up and all groups were done under the same conditions in the experiment.

### Hypoxia/reoxygenation treatment to cardiomyocytes

The hypoxia/reoxygenation model was established according to our previously published method, with few modifications^6,25,26^. We started experiments after being cultured for 48–72 h and pretreated with different agents. For simulation of ischemia, the cardiomyocyte medium was replaced with the DMEM without glucose and serum, which was flushed with a gas mixture (95% N2 and 5% CO_2_) for 15 min. The cardiomyocytes were incubated in a modular incubator chamber with a gas mixture of 95% N2 and 5% CO_2_ at 37 °C for 4 h. After hypoxia incubation, the cells were provided with a serum-free medium and then restored to 95% air and 5% CO_2_ to reoxygenation for 8 h.

### Cell viability and lactate dehydrogenase (LDH) release assay

Cell viability was assessed by using a Cell Counting Kit-8 (CCK-8) (Dojindo Laboratories, CK04) according to the manufacturer's protocol^27,28^. About 0.5 × 10^4^ cells were seeded onto 96-well plates and then pretreated with different agents. Following hypoxia/reoxygenation exposure, 10 μl CCK-8 was added to each well. Then the cells were incubated at 37 °C for 1 h. After incubation, the OD value at 450 nm was determined using an Inf inite® 200 Microplate Reader (Tecan, M200).

Lactate dehydrogenase (LDH) release in the extracellular medium was detected using an LDH assay kit (Roche, Mannheim, Germany) according to the manufacturer's instructions.

### Quantitative real-time PCR

Total RNA was extracted from cultured cardiomyocytes with Trizol reagent (Invitrogen, Green Island, NY). Reverse transcription was performed with ReverTra Ace reverse transcriptase (Toyobo, Osaka, Japan) according to the manufacturer's instructions. The transcript level of mRNA was determined with the Bio-Rad CFX96 Real-time PCR system-C1000 Thermal Cycler (Bio-Rad Laboratories, Hercules, CA, USA). Each real-time PCR (20 μl total volume) contained 1 μl of template DNA (100 mg) or cDNA, 10 μl Ex Taq, 1 μl of each of the forward and reverse primers, and 7 μl of sterile deionized water. The samples were denatured by heating at 95 °C for 3 min, followed by 39 cycles of amplification and quantification (95 °C for 15 s and 58 °C for 15 s, respectively) and by a final extension cycle (72 °C, 90 s). The primers used for amplification were (from 5'to 3'): CHOP-F: TATCTCATCCCCAGGAAACG, CHOP-R:CTGCTCCTTCTCCTTCATGC, Grp78-F:TTCAGCCAATTATCAGCAAACTCT, Grp78-R:TTTTCTGATGTATCCTCTTCACCAGT, sXbp-1-F:CCTTGTGGTTGAGCAGAAC, sXbp-1-R:CCTGCACCTGCTGCGGAC, GAPDH-F:ACCCCAGTTTACTCCATCCC, GAPDH-R:TGTTCCGGGTGGTTCTGCAG. The mRNA levels were quantified first against GAPDH levels in the same sample, then normalized to the control group, set up as onefold. These experiments were repeated six times.

### Western blot analysis

Neonatal mice cardiomyocytes were cultured at a cell density of 1.2 × 10^6^ in 60-mm plastic culture dishes. Western blot analysis^7,29^ was performed on cultured cardiomyocytes from the different groups. After being washed twice with the ice-cold PBS, the cells were lysed at 4 °C for 10 min with RIPA buffer (Beyotime, Nanjing, China) containing a cocktail of protease inhibitors (Roche, USA), then centrifuged at 20,000×*g* and 4 °C for 20 min. To analyze the mitochondrial and cytosol cytochrome c protein levels respectively, the mitochondrial extracts were isolated from the cultured cardiomyocytes using the Mitochondria Isolation Kit (Beyotime) according to our previous experiments^8^. COX IV was used as the loading control for mitochondrial fractions and GAPDH was used as the loading control for cytosol fractions and total cellular. Next, protein concentration was determined using the BCA Protein Assay Kit (Beyotime, Nanjing, China), and all samples were boiled at 97 °C for 10 min to inactivate the proteins. After that, equal amounts of protein (50 µg/slot) were separated on an 8–12%SDS-PAGE gel and transferred onto an NC membrane (Millipore, MA), which was blocked with 5% milk Tris-buffered saline-tween 20, incubated with primary antibodies (1:1000) at 97 °C overnight, and then second antibodies (1:1000) for 1 h. The following primary antibodies were used for the western blots: mouse anti-CHOP/GADD153 (CST, #2895), mouse monoclonal anti–cytochrome c (Abcam, ab60256), mouse anti-caspase-3 (Santa Cruz Biotechnology, sc-56053), Rabbit anti-cleaved caspase-3 (Cell Signaling Technology, #9661), Rabbit anti-COX IV (Cell Signaling Technology, #4844), Rabbit anti-AMPKα (Santa, SC-23792), Rabbit anti- Bip/GRP78 (Beyotime, AB310), Rabbit anti-Phospho-AMPKα (Thr172) (Cell Signaling Technology, #2535), mouse anti-eIF2α (Abcam, QB-5369), Rabbit anti-Phospho-eIF2α (Cell Signaling Technology, #9721), and anti-rabbit GAPDH (Cell Signaling Technology #5174). Densitometric analyses were performed using an Odyssey Infrared Imaging System (LI-COR).

### Detection of apoptotic cardiomyocytes

Terminal deoxynucleotidyl transferase dUTP nick-end labeling (TUNEL) was used to detect the apoptotic cardiomyocytes according to our previous experiments by using an in situ cell death detection kit (Roche, USA)^6,30,31^. After being rinsed twice for ice PBS, the cardiomyocytes slides were fixed in 4% paraformaldehyde at room temperature for 30 min, followed by permeabilization with 0.1% TritonX-100 in 0.1% sodium citrate for 10 min at room temperature. Then, a 50 μl TUNEL reaction mixture was added to the sample and incubated in a humidified chamber for 60 min. DAPI was used to label nuclei. After cardiomyocytes were mounted in a mounting medium, apoptotic cells images were obtained using a Zeiss confocal laser scanning microscope (Carl Zeiss, LSM 510 Meta Confocal Laser Scanning Microscope).

Apoptosis was examined to determine the protective effect of irisin using a FITC-labeled Annexin V/propidium iodide (PI) Apoptosis Detection kit (BD Biosciences, Franklin Lakes, NJ) following the manufacturer's instructions. Briefly, adherent cardiomyocytes were enzymatically digested for 50 s with 0.25% trypsin and collected together with floating dead cells. Cardiomyocytes were then incubated with Annexin V (1:20) for 3 min followed by PI for 15 min in the dark, at room temperature. The cells were subjected to flow cytometry on a BD FACS Calibur, and the data were analyzed using FlowJosoftware. Cells in the early stages of apoptosis were Annexin V positive, whereas cells that were both PI and Annexin V positive were in the later stages of apoptosis. Apoptotic cells were expressed as a percentage of the total number of cells.

### Statistical analysis

All experimental data are expressed as the mean ± standard error of the mean (SEM), and each experiment was performed a minimum of 3 times. Raw data were analyzed using GraphPad Prism 5.0 software (GraphPad Software, Inc., San Diego, CA, USA). Data were also analyzed using a one-way analysis of variance(ANOVA). A two-tailed *P* < 0.05 was taken to indicate a statistically significant difference.

### Ethics statement

This study was performed strictly with the recommendations in the Guide for the Care and Use of Laboratory Animals of the National Institutes of Health. The protocol was approved by the Animal Ethical and Welfare Committee of North Sichuan Medical College. This study was approved by the Medical Ethics Committee.

## Result

### The protective effects of irisin on H/R-injury

**Figure 1:**
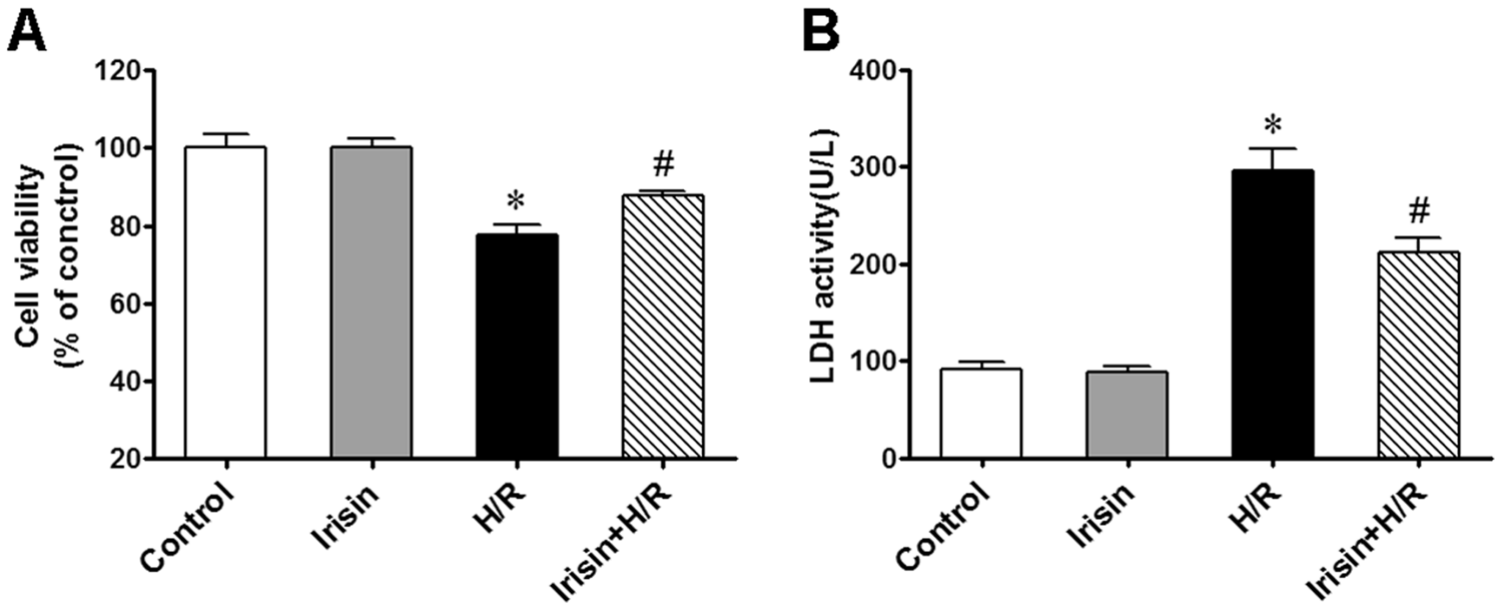
Irisin attenuated H/R induced cell injury. Cell viability was determined by CCK-8 kit, pretreated with irisin(100 ng/ml) significantly alleviated cell viability decrease induced by H/R exposure (**A**). Irisin pre-treatment also significantly alleviated the release of LDH induced by H/R exposure (**B**). *P < 0.05 versus control group, ^*#*^*P* < 0.05 versus H/R group, n = 6.

The percentage of survived cardiomyocytes was proportional to the measured cell viability^32^. Here we evaluated the effect of irisin on cell viability to investigate its potential protective effect on cardiomyocytes undergoing H/R injury. For that, cardiomyocytes pretreated with irisin underwent H/R treatment. We found that pre-treatment with irisin could successfully attenuate the decrease in cell viability caused by the H/R treatment (Fig. 1A). Moreover, cell injury was quantified by measuring the LDH levels in the extracellular medium. Irisin pre-treatment significantly reduced the LDH release in H/R-treated cardiomyocytes (Fig. 1B), demonstrating the protective effects of irisin against H/R-injury.

### Irisin reduces H/R-induced apoptosis of cultured cardiomyocytes

**Figure 2:**
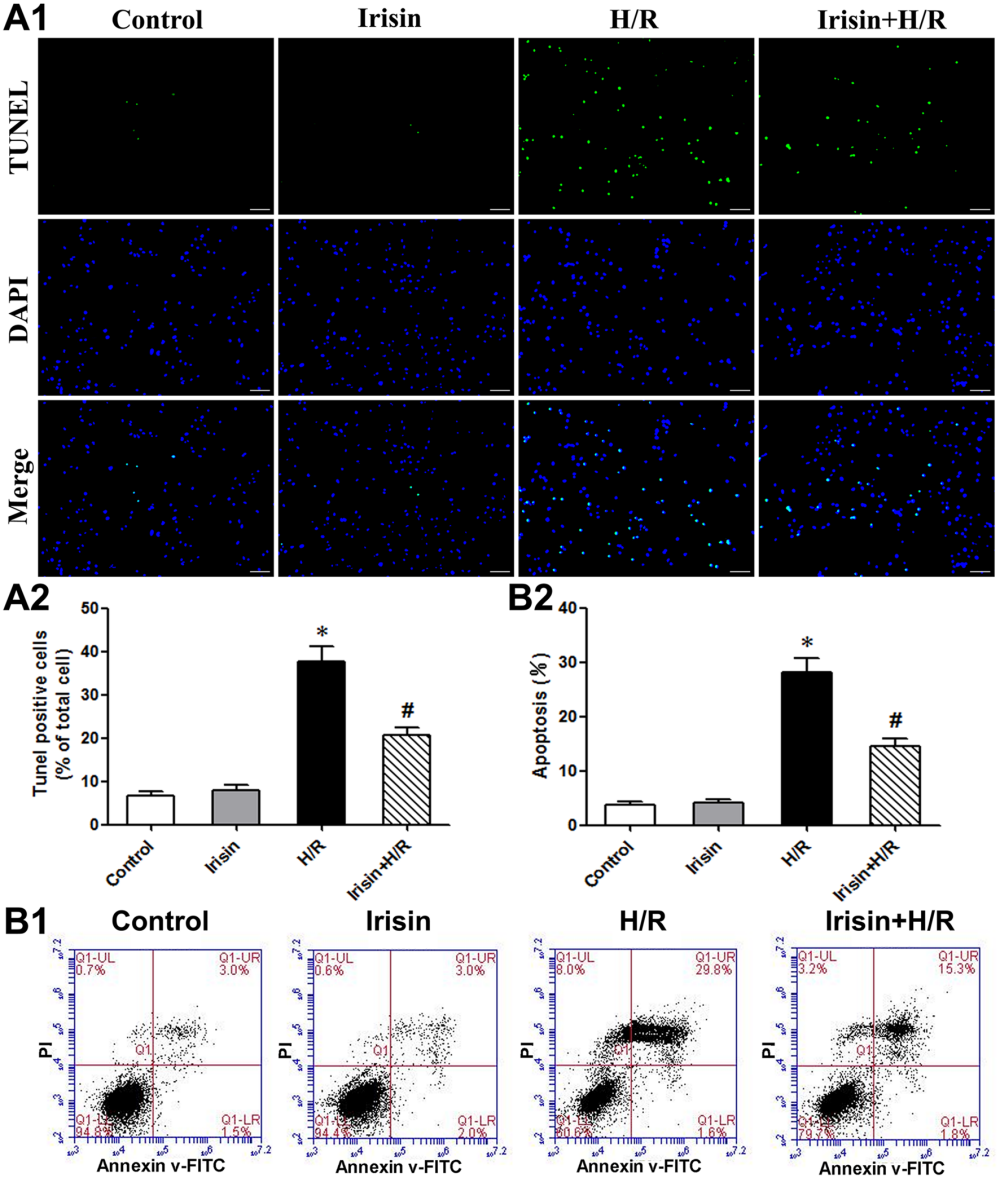
Irisin attenuated H/R-induced cardiomyocyte apoptosis. Representative images (**A1**) and quantification (**A2**) of cardiomyocytes in situ TUNEL assay. TUNEL-positive is green, and DAPI is blue. Scale bar: 200 mm. (**B**) Flow cytometry analysis of Annexin-V FITC and propidium iodide staining in cardiomyocytes. *P < 0.05 versus control group, ^*#*^*P* < 0.05 versus H/R group, n = 6.

We investigated the effect of irisin on the H/R-induced apoptosis in cultured cardiomyocytes (Fig. 2A). More TUNEL positive cells (green) were observed in H/R-treated cultures (Fig. 2A1). Quantitative analysis also revealed that H/R increased TUNEL-positive apoptotic cells compared to cells under regular conditions. The pre-treatment with irisin reduced the number of TUNEL-positive cells compared to the H/R group (Fig. 2A2). The Annexin V/PI analysis results with flow cytometry confirmed that irisin reduced H/R-induced apoptosis (Fig. 2B).

### Irisin blocks the mitochondrial apoptotic pathway

**Figure 3:**
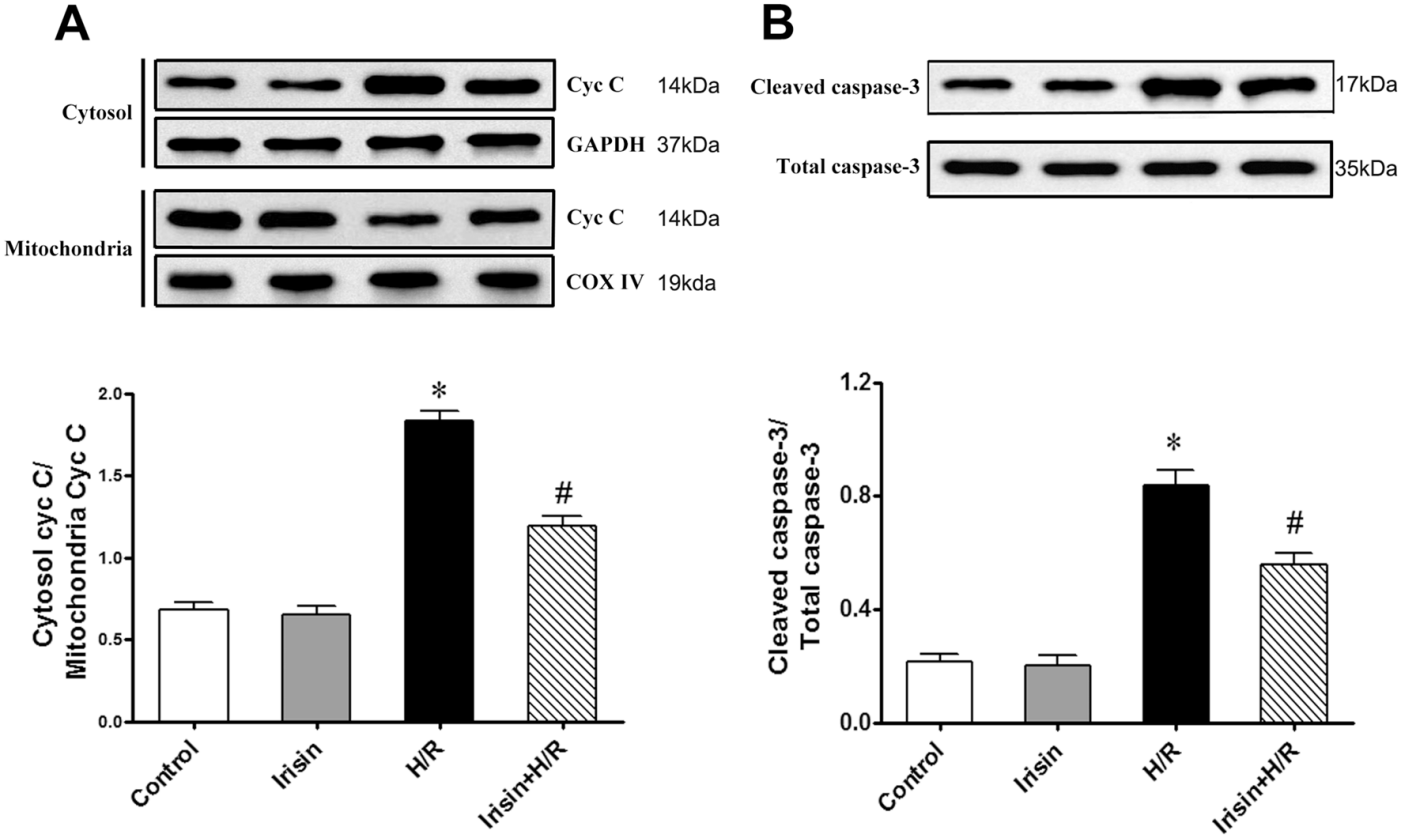
Irisin inhibited H/R-induced cytochrome c release and caspase-3 activation in cardiomyocytes. (**A**) Assessment of cytochrome c (Cyt c) in mitochondria and cytosol derived from cardiomyocytes subjected to H/R. COX IV: Cytochrome c oxidase subunit IV; GAPDH: glyceraldehyde-3-phosphate dehydrogenase. (**B**) Caspase-3 activation in cardiomyocytes subjected to H/R. *P < 0.05 versus control group, ^*#*^*P* < 0.05 versus H/R group, n = 6.

Mitochondrial dysfunction may initiate apoptosis by releasing pro-apoptotic factors, such as cytochrome c, from the mitochondrial intermembrane space into the cytoplasm to trigger apoptosis via a caspase-3-dependent pathway. Accordingly, changes in mitochondrial and cytosol cytochrome c were measured by immunoblotting. As shown in Fig. 3A, H/R treatment caused the release of cytochrome c from mitochondria, and irisin administration could reduce the release of cytochrome c from mitochondria to the cytosol. The effect of irisin in preventing apoptotic cell death was evident in the immunoblotting of caspase 3, a key enzyme involved in the execution of apoptosis. Cardiomyocytes with H/R treatment showed a significant increase in caspase-3 activity. In contrast, pre-treatment with irisin significantly inhibited the H/R-induced activation of caspase-3 (Fig. 3B), suggesting that the irisin-reduced H/R-induced apoptosis was mediated through the blockage of caspase-3-dependent cardiomyocytes apoptosis.

### H/R resulted in excessive ER stress and CHOP upregulation

**Figure 4:**
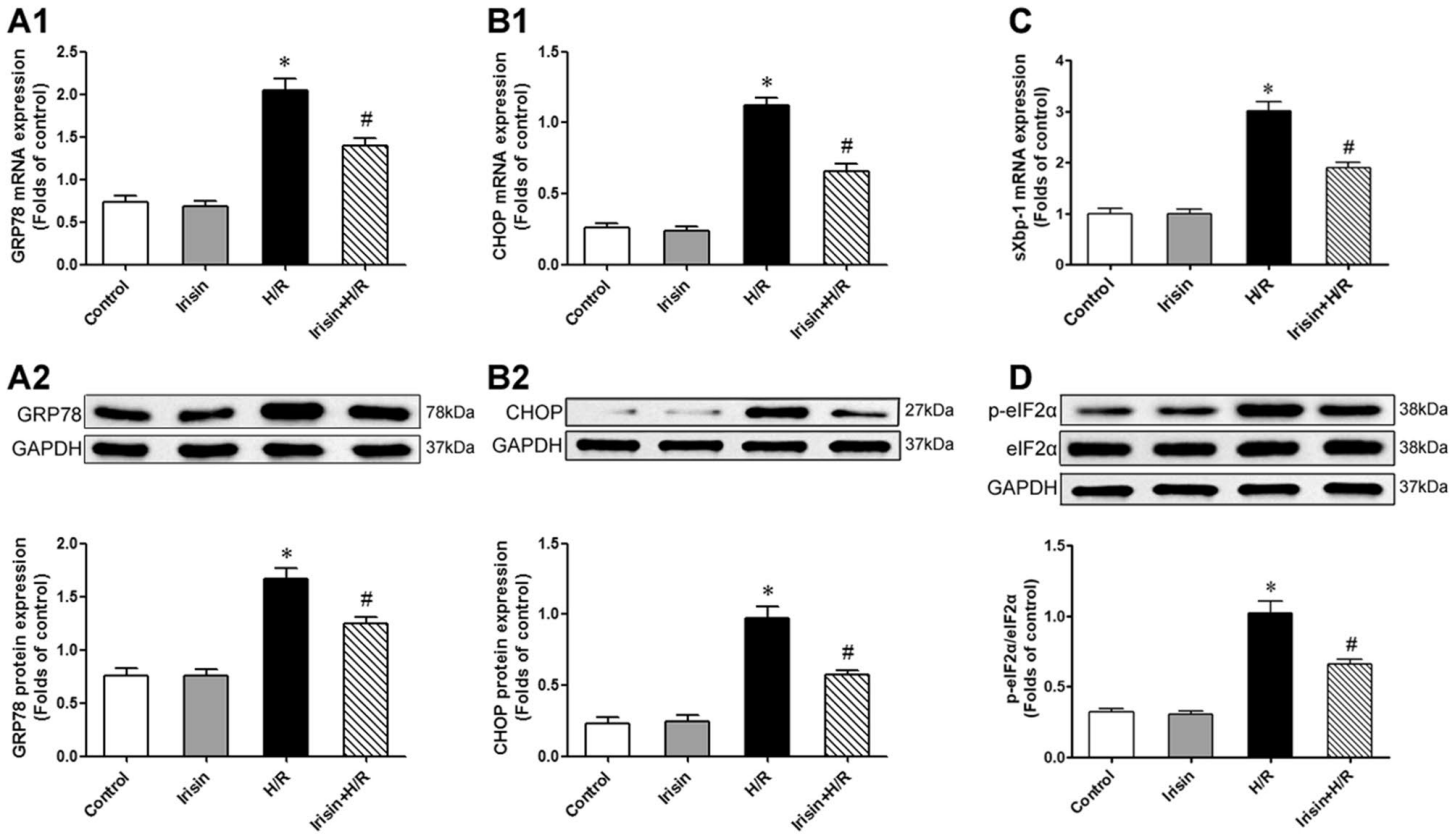
H/R treatment resulted in excessive ER stress and CHOP upregulation. The GRP78, CHOP, sXbp-1, and eIF2α expressions were assessed by PCR or Western blot analysis. (**A**) Effects of irisin on H/R induced GRP78 mRNA (**A1**) and protein (**A2**) expression. (**B**) Effects of irisin on H/R induced CHOP mRNA (**B1**) and protein (**B2**) expression. (**C**) Effects of irisin on H/R induced sXbp-1 mRNA expression. (**D**) Effects of irisin on H/R induced eIF2α phosphorylation. *P < 0.05 versus control group, ^*#*^*P* < 0.05 versus H/R group, n = 6.

GRP78/BiP is a marker of ER stress. Excessive ER stress results in the over-expression of GRP78 and thus leads to upregulation of pJNK, Caspase-12, and CHOP, which could induce cell apoptosis^33,34^. In our experiment, H/R induced a significant upregulation of GRP78, and this effect could be attenuated by irisin pre-treatment (Fig. 4A). Consistently with this phenomenon, CHOP expression in the H/R group was significantly up-regulated compared to the control and irisin groups. On the other hand, irisin pre-treatment showed the ability to reduce CHOP expression induced by H/R (Fig. 4B). Meanwhile, we find that the level of spliced Xbp-1 mRNA in cardiomyocytes was increased in H/R-treated cells and irisin significantly reversed the increase(Fig. 4C). The phosphorylation level of eIF2α was increased in H/R-treated cardiomyocytes compared to the control group, whereas irisin ameliorated these changes(Fig. 4D).

### Irisin blunted H/R induced apoptosis via an AMPK-mediated pathway

**Figure 5:**
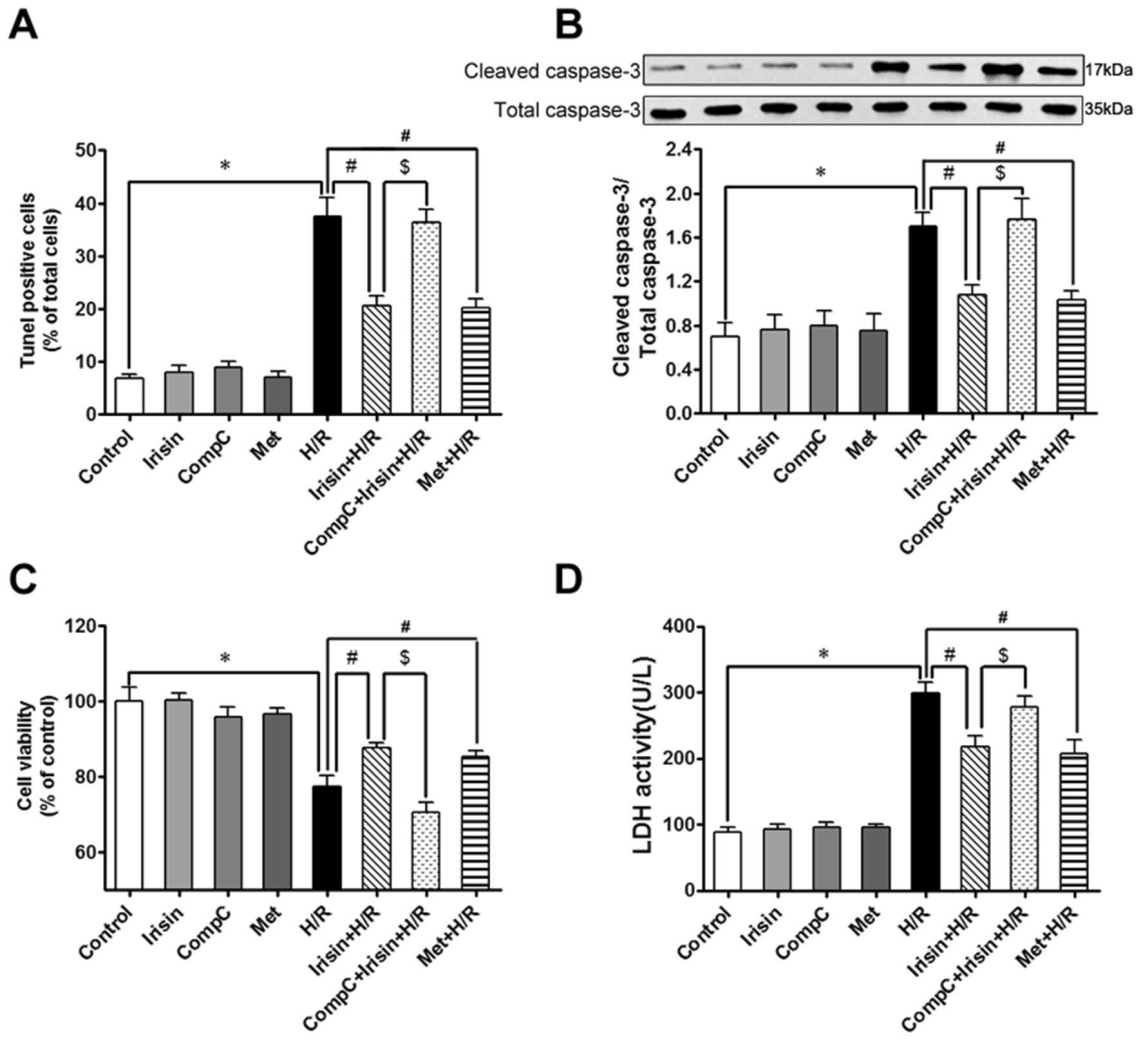
Irisin blunted H/R induced apoptosis via an AMPK-mediated manner. To investigate the effect of the AMPK signal pathway in cardioprotection of irisin, the AMPK inhibitor compound C was used to pretreat cardiomyocytes(20 μM, 1 h) before irisin pre-treatment, and the AMPK activator metformin pretreats (2.5 mM, 1 h) group was used to make a comparison of apoptosis (**A**), caspase-3 activities (**B**), cell viability (**C**), and LDH activity (**D**). *P < 0.05 versus control group, *#P* < 0.05 versus H/R group, $*P* < 0.05 versus Irisin + H/R group, n = 6.

Notably, the percentage of TUNEL-positive cells to total cells in the H/R group increased significantly compared to the control group. The pre-treatment partly blunted apoptosis with either irisin or AMPK-activator metformin (Fig. 5A). Consistent with the change of the apoptotic index in different groups, the activity of caspase-3 in the H/R group significantly increased compared to the control group, and this change was attenuated by either irisin or metformin pre-treatment (Fig. 5B). Similarly, cell viability in the H/R group significantly decreased compared to the control group, and this change was attenuated by either irisin or metformin pre-treatment (Fig. 5C). Moreover, LDH levels of the extracellular medium in the H/R group significantly increased compared to the control group, and this change was attenuated by either irisin or metformin pre-treatment (Fig. 5D). The effect of irisin on the apoptotic index (Fig. 5A), caspase-3 activity (Fig. 5B), cell viability (Fig. 5C), and LDH release (Fig. 5D) in the H/R group was abolished by AMPK-inhibitor Compound-C pre-treatment.

### H/R resulted in AMPK inactivation, and irisin showed similar effects on H/R cardiomyocytes as metformin

**Figure 6:**
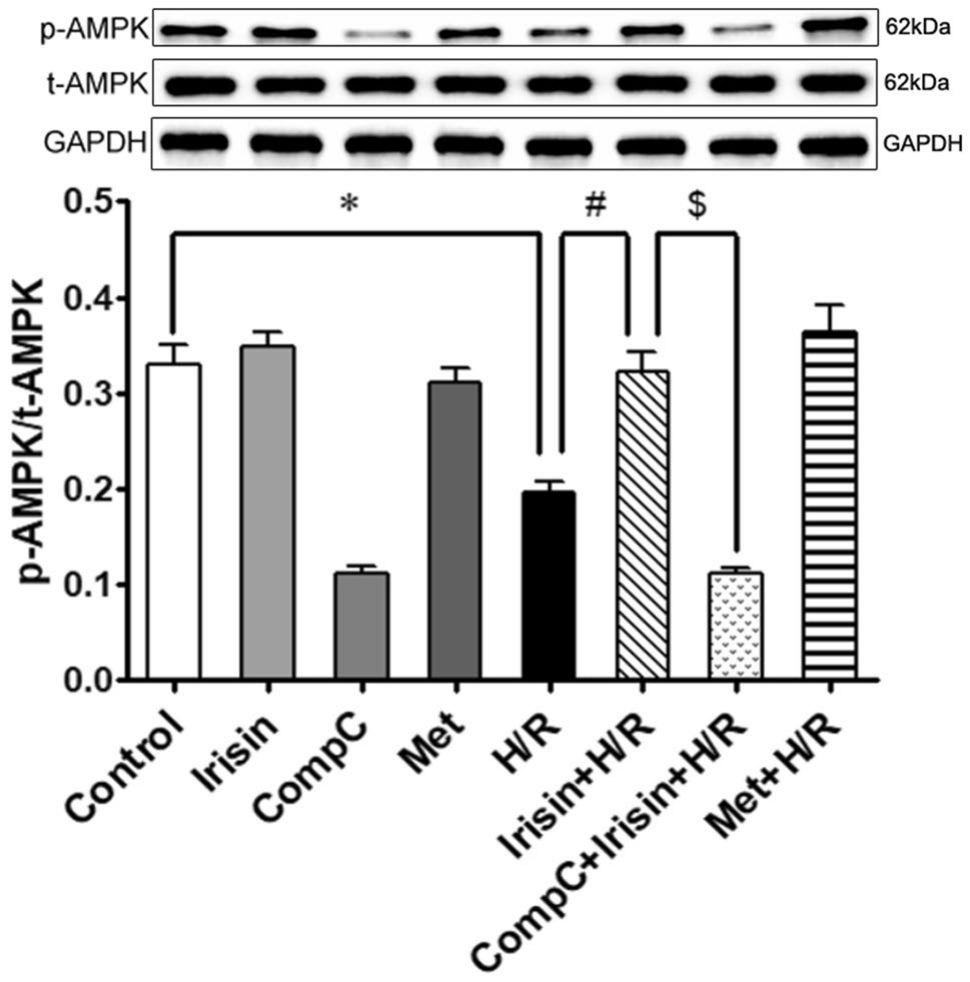
H/R treatment led to AMPK inactivation, and irisin showed the same effects on H/R cardiomyocytes as metformin. AMPK activation was estimated by detecting the phosphorylated AMPK. Irisin attenuated the inactivating of AMPK induced by H/R exposure and compound C pretreat abolished this effect. *P < 0.05 versus control group, ^*#*^*P* < 0.05 versus H/R group, ^$^*P* < 0.05 versus Irisin + H/R group, n = 6. *p-AMPK* phosphorylated AMPK, *t-AMPK* total AMPK.

As an energy metabolism-regulating kinase, AMPK is activated by either increasing its phosphorylation or by up-regulating the expression of total AMPK^35^. Expression of total AMPK and phosphorylated AMPK was detected using western blot (Fig. 6). In our experiment, there was an evident decrease in phosphorylated AMPK expression in the H/R group compared to the control group, and there was no difference in the expression of total AMPK between the different groups. Irisin had the same effect on increasing AMPK phosphorylation in cardiomyocytes exposure to H/R as AMPK activator metformin, and this phenomenon could be obliterated by AMPK-inhibitor Compound-C pre-treatment.

### Irisin showed its protective effects against H/R injury-induced ER stress in an AMPK-dependent manner

**Figure 7:**
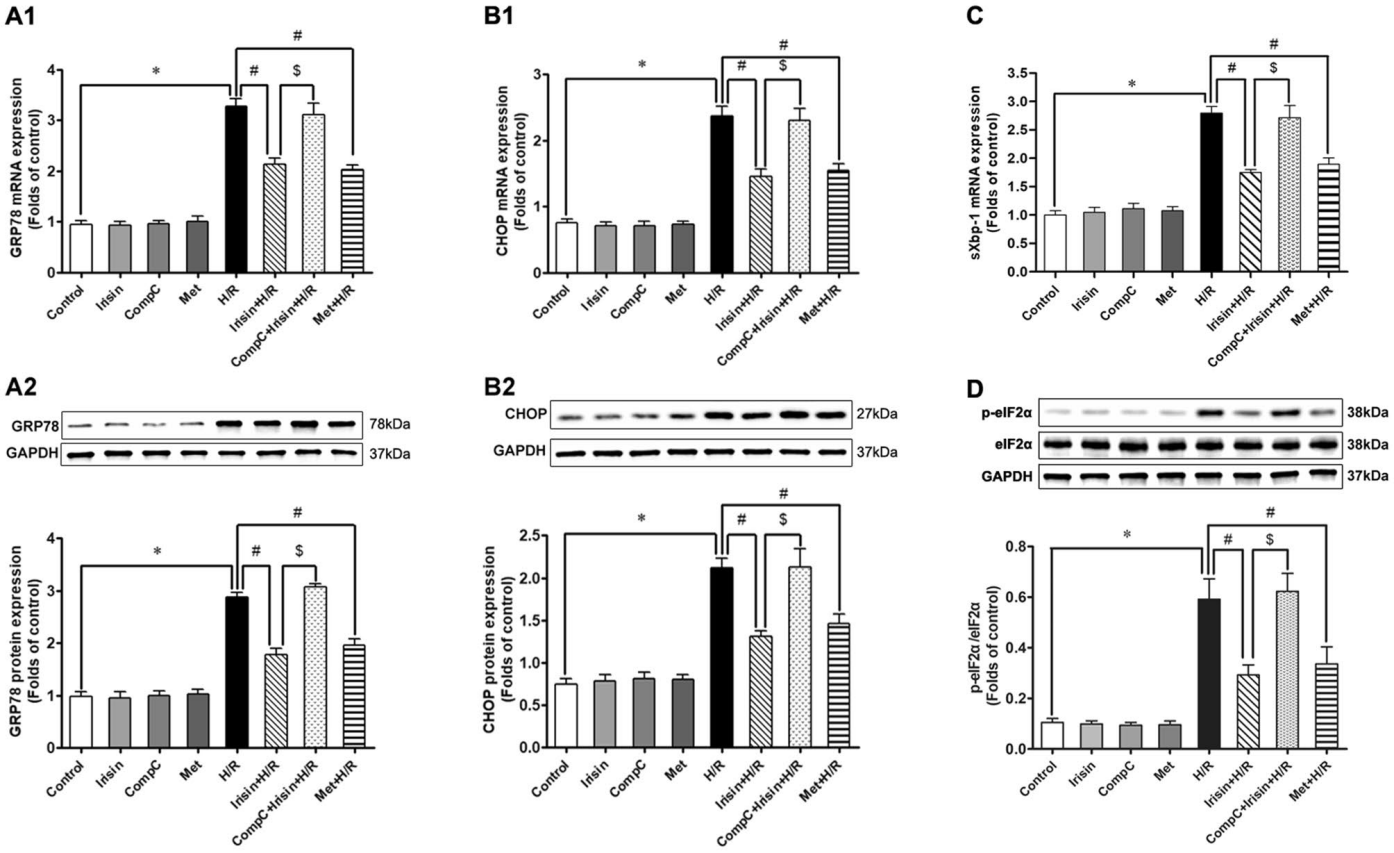
Irisin showed its protective effects against H/R injury-induced ER stress in an AMPK-dependent manner. AMPK activator metformin showed the same effect as irisin in attenuating ER stress induced by H/R exposure. AMPK potent inhibitor compound C pre-treatment abolished cardioprotection of irisin on H/R cardiomyocytes. *P < 0.05 versus control group, ^*#*^*P* < 0.05 versus H/R group, ^$^*P* < 0.05 versus Irisin + H/R group, n = 6.

To investigate whether AMPK associated signaling pathways were involved in irisin's protective effects, cardiomyocytes were pretreated with AMPK inhibitor compound C before irisin pre-treatment and exposure to H/R. Then, we compared the expression of GRP78, CHOP, sXbp-1, and the phosphorylation of eIF2α between different groups to explore the underlying relationship among irisin, ER stress, and AMPK signaling pathways. There was no difference in GRP78, CHOP, and sXbp-1 expression and eIF2α phosphorylation among groups not exposed to H/R. As depicted in Fig. 7A, irisin had the same effect as AMPK activator metformin in decreasing the GRP78 expression compared to the H/R group, and compound C pre-treatment could abrogate this protection. In line with the change of GRP78, CHOP expression was significantly up-regulated in the H/R group, irisin or metformin pre-treatment decreased CHOP expression levels compared to the H/R group (Fig. 7B). sXbp-1 expression was significantly up-regulated in the H/R group, irisin or metformin pre-treatment decreased sXbp-1 expression levels compared to the H/R group (Fig. 7C). The phosphorylation of eIF2α was significantly up-regulated in the H/R group, irisin or metformin pre-treatment decreased eIF2α phosphorylation levels compared to the H/R group (Fig. 7D). Finally, we observed that compound C pre-treatment markedly blocked irisin's effect on cardiomyocytes undergoing H/R.

## Discussion

In the present study, by using a stimulated hypoxia/reoxygenation model, we verified that apoptosis was essential to H/R injury, and H/R injury-induced apoptosis was closely associated with ER stress. Moreover, we also demonstrated that ER stress was, at least partly, regulated by AMPK. We first showed that irisin protected cardiomyocytes from H/R injury via attenuating ER stress in an AMPK-dependent manner. Therefore, AMPK-mediated ER stress might be a new target pathway for exploring pharmacological treatment for I/R-induced myocardium injury^9,36,37^.

ER stress is an innate adaptive mechanism existing in different species. Mild ER stress presents a protective effect against injury factors. In contrast, prolonged stress shows lethal damage to cells or organs^33,38^. In mild ER stress, GRP78 (GRP78, glucose-regulated protein 78) released its transducers, including IRE-1, PERK, ATF-6, and allow them to dimerize activation or move to other locations, then triggering the downstream signal pathways to hold ER homeostasis by decreasing the accumulating of unfolded proteins and promoting unfolded proteins to fold. There is a conversion from pro-surviving pathways to the pro-apoptotic pathways under severe injury in different degrees of stress^39^. ER stress-mediated intrinsic apoptosis pathways involve several proteins, including the transcription factor CHOP, phosphorylated JNK, and the ER-resident cysteine protease caspase-12^33^. Besides, ER stress-associated apoptosis is characterized by upregulation of CHOP, increased phosphorylated JNK, and cleaved caspase-12^40^. Each of them could activate the downstream pro-apoptotic signal transducers, leading to caspase-3 activation and terminal apoptosis.

In our experiment, we detected the expression of GRP78 and CHOP to investigate the influence of irisin on H/R-induced ER stress. Western blot revealed that H/R exposure induced upregulation of GRP78 compared to the control group, suggesting that H/R injury induces excessive ER stress. In addition, increased CHOP expression confirmed that H/R injury activated ER stress-mediated CHOP apoptotic signal. Studies demonstrated that ER stress-associated apoptosis is closely related to both H/R injury and CHOP is usually considered downstream of ER stress-induced apoptosis^11,40,41^. Furthermore, Runtao et al.^42^ showed that ischemia/reperfusion injury induces excessive ER stress and activates the downstream pro-apoptotic signal pathways. Therefore, ischemic post-conditioning could protect the myocardium from I/R injury by attenuating ER stress-associated apoptosis. Here, we show for the first time that irisin could protect cardiomyocytes against H/R injury by attenuating ER stress.

As an energy metabolic kinase, AMPK activation significantly reduced the percentage of apoptotic cardiomyocytes and the activity of the apoptotic effectors of ER stress in a cardioplegia-induced H/R injury model^9^. Shintani et al.^43^ also found that nonfatal damage to cells could reduce the energy consumption and AMPK activation, which shows a protective effect on cell injury via increasing stress tolerance. Additionally, using a heart failure model in dogs, Sasaki and Asanuma^44^ discovered that metformin and another AMPK activator (AICAR) have equivalent effects of decreasing cardiomyocytes apoptosis and improving cardiac function in failing dog hearts. Interestingly, treatment with compound C inhibited the effects of metformin and AICAR, suggesting the primary role of AMPK activation in reducing apoptosis and preventing heart failure. Therefore, AMPK might be a new target for pharmacological potential in reducing cardiomyocyte H/R injury or myocardium I/R injury^36^. Although there is much evidence about the protective effect of AMPK activation, there was no research suggesting that irisin could protect the myocardium by activating AMPK and attenuating ER stress.

AMPK activator metformin and AMPK inhibitor compound C was used in our experiment to investigate the possible role of AMPK in the cardioprotection by irisin. Our experiment testified that irisin has the same effect as metformin to activate AMPK and decrease the expression of GRP78 and CHOP in a stimulated H/R model. Our results showed that compared to the compound C pre-treatment group, irisin's effects on H/R cardiomyocytes disappeared, suggesting that the cardioprotection of irisin was partly mediated by AMPK activation and AMPK might be an upstream regulator of ER stress and ER stress-associated apoptosis. The beneficial effects of irisin's activation of AMPK-related pathways on cells have been widely explored. In the human placenta, irisin can induce trophoblast differentiation via AMPK activation and improve trophoblast functions^45^. In addition, irisin also protects against pressure overload-induced cardiac hypertrophy via activating AMPK-ULK1 signaling^46^. In diabetic mice, irisin attenuates myocardial ischemia/reperfusion injury through the AMPK pathway^47^. Similarly, in the setting of IR injury and hyperglycemia stress, irisin can increase the survival rate of cardiomyocytes by activating the AMPK pathway^48^. Our study suggests that AMPK was responsible for mitochondrial dysfunction-induced energy metabolic disorders and involved in regulating ER stress signal pathway. However, whether irisin activates AMPK directly or through some intermediate receptor, has not yet been determined. Previous study has shown that irisin can downregulate ATP levels in HSMCs, triggering AMPK phosphorylation^49^. The majority of ATP is generated in mitochondria by oxidative phosphorylation. Uncoupling proteins on the mitochondria can release the coupling relationship between oxidation and phosphorylation in part of the normal respiratory chain, hindering the normal production of ATP. Studies have confirmed that interaction between irisin and UCP2(an uncoupling protein) allows for the prevention of IR-induced injury to the lung via improvement of mitochondrial function^50^. Besides, irisin can increase the ROS level in L6 cells, and using the ROS scavenger resulted in a decrease in AMPK phosphorylation^51^. Their research further demonstrated that calcium may play an upstream of ROS in irisin-mediated signaling. These results indicate that irisin increases AMPK phosphorylation through the Ca^2+^/ROS pathway in the skeletal muscles. It is worth noting that cardiac muscle exercise produces more irisin than skeletal muscle^52^. Importantly, the study revealed the presence of yet-to-be-identified irisin receptor(s) on the H_9_C_2_ cell membrane^53^. Through this receptor, irisin may activate several downstream signaling pathways, such as the PI3K-AKT pathway. Similarly, they found that irisin also can increase Ca^2+^ in cardiomyocytes. However, they did not verify the above experimental results of irisin in animal models.

## Conclusion

In conclusion, our data revealed that ER stress plays an essential role in cell death during H/R and that AMPK activation is protective. Irisin has a potent effect in protecting cardiomyocytes from H/R injury by significantly attenuating excessive ER stress-induced apoptosis in an AMPK-mediated manner.

## Data Availability

All data generated or analyzed during this study are included in this published article.

## References

[1] Virani SS, Alonso A, Benjamin EJ, Bittencourt MS, Callaway CW, Carson AP (2020). Heart disease and stroke statistics-2020 update: A report from the American Heart Association. Circulation.

[2] Sanchis D, Llovera M, Ballester M, Comella JX (2008). An alternative view of apoptosis in heart development and disease. Cardiovasc. Res..

[3] Takemura G, Kanoh M, Minatoguchi S, Fujiwara H (2013). Cardiomyocyte apoptosis in the failing heart: A critical review from definition and classification of cell death. Int. J. Cardiol..

[4] Wu MY, Yiang GT, Liao WT, Tsai AP, Cheng YL, Cheng PW (2018). Current mechanistic concepts in ischemia and reperfusion injury. Cell. Physiol. Biochem..

[5] Binder A, Ali A, Chawla R, Aziz HA, Abbate A, Jovin IS (2015). Myocardial protection from ischemia-reperfusion injury post coronary revascularization. Expert Rev. Cardiovasc. Ther..

[6] Yue R, Hu H, Yiu KH, Luo T, Zhou Z, Xu L (2012). Lycopene protects against hypoxia/reoxygenation-induced apoptosis by preventing mitochondrial dysfunction in primary neonatal mouse cardiomyocytes. PLoS ONE.

[7] Luo T, Yue R, Hu H, Zhou Z, Yiu KH, Zhang S (2015). PD150606 protects against ischemia/reperfusion injury by preventing mu-calpain-induced mitochondrial apoptosis. Arch. Biochem. Biophys..

[8] Yue R, Xia X, Jiang J, Yang D, Han Y, Chen X (2015). Mitochondrial DNA oxidative damage contributes to cardiomyocyte ischemia/reperfusion-injury in rats: Cardioprotective role of lycopene. J. Cell. Physiol..

[9] Yeh CH, Chen TP, Wang YC, Lin YM, Fang SW (2010). AMP-activated protein kinase activation during cardioplegia-induced hypoxia/reoxygenation injury attenuates cardiomyocytic apoptosis via reduction of endoplasmic reticulum stress. Mediators Inflamm..

[10] Matsui Y, Takagi H, Qu X, Abdellatif M, Sakoda H, Asano T (2007). Distinct roles of autophagy in the heart during ischemia and reperfusion: Roles of AMP-activated protein kinase and Beclin 1 in mediating autophagy. Circ. Res..

[11] Doroudgar S, Glembotski CC (2013). New concepts of endoplasmic reticulum function in the heart: Programmed to conserve. J. Mol. Cell. Cardiol..

[12] Ray D, Mukherjee S, Falchi M, Bertelli A, Das DK (2010). Amelioration of myocardial ischemic reperfusion injury with *Calendula officinalis*. Curr. Pharm. Biotechnol..

[13] Lee YM, Chen HR, Hsiao G, Sheu JR, Wang JJ, Yen MH (2002). Protective effects of melatonin on myocardial ischemia/reperfusion injury in vivo. J. Pineal Res..

[14] Xu J, Hu H, Chen B, Yue R, Zhou Z, Liu Y (2015). Lycopene protects against hypoxia/reoxygenation injury by alleviating ER stress induced apoptosis in neonatal mouse cardiomyocytes. PLoS ONE.

[15] Zhu D, Wang H, Zhang J, Zhang X, Xin C, Zhang F (2015). Irisin improves endothelial function in type 2 diabetes through reducing oxidative/nitrative stresses. J. Mol. Cell. Cardiol..

[16] Park MJ, Kim DI, Choi JH, Heo YR, Park SH (2015). New role of irisin in hepatocytes: The protective effect of hepatic steatosis in vitro. Cell. Signal..

[17] Lee P, Linderman JD, Smith S, Brychta RJ, Wang J, Idelson C (2014). Irisin and FGF21 are cold-induced endocrine activators of brown fat function in humans. Cell Metab..

[18] Jeremic N, Chaturvedi P, Tyagi SC (2017). Browning of white fat: Novel insight into factors, mechanisms, and therapeutics. J. Cell. Physiol..

[19] Luo Y, Qiao X, Ma Y, Deng H, Xu CC, Xu L (2020). Disordered metabolism in mice lacking irisin. Sci. Rep..

[20] Lu J, Xiang G, Liu M, Mei W, Xiang L, Dong J (2015). Irisin protects against endothelial injury and ameliorates atherosclerosis in apolipoprotein E-Null diabetic mice. Atherosclerosis.

[21] Bostrom P, Wu J, Jedrychowski MP, Korde A, Ye L, Lo JC (2012). A PGC1-alpha-dependent myokine that drives brown-fat-like development of white fat and thermogenesis. Nature.

[22] Villarroya F (2012). Irisin, turning up the heat. Cell Metab..

[23] Perez-Sotelo D, Roca-Rivada A, Baamonde I, Baltar J, Castro AI, Dominguez E (2017). Lack of adipocyte-Fndc5/irisin expression and secretion reduces thermogenesis and enhances adipogenesis. Sci. Rep..

[24] Lokuta A, Kirby MS, Gaa ST, Lederer WJ, Rogers TB (1994). On establishing primary cultures of neonatal rat ventricular myocytes for analysis over long periods. J. Cardiovasc. Electrophysiol..

[25] Liu CL, Li X, Hu GL, Li RJ, He YY, Zhong W (2012). Salubrinal protects against tunicamycin and hypoxia induced cardiomyocyte apoptosis via the PERK-eIF2alpha signaling pathway. J. Geriatr. Cardiol..

[26] Guo J, Bian Y, Bai R, Li H, Fu M, Xiao C (2013). Globular adiponectin attenuates myocardial ischemia/reperfusion injury by upregulating endoplasmic reticulum Ca(2)(+)-ATPase activity and inhibiting endoplasmic reticulum stress. J. Cardiovasc. Pharmacol..

[27] Pi H, Xu S, Zhang L, Guo P, Li Y, Xie J (2013). Dynamin 1-like-dependent mitochondrial fission initiates overactive mitophagy in the hepatotoxicity of cadmium. Autophagy.

[28] Xu SC, Chen YB, Lin H, Pi HF, Zhang NX, Zhao CC (2012). Damage to mtDNA in liver injury of patients with extrahepatic cholestasis: The protective effects of mitochondrial transcription factor A. Free Radical Biol. Med..

[29] Grall S, Prunier-Mirebeau D, Tamareille S, Mateus V, Lamon D, Furber A (2013). Endoplasmic reticulum stress pathway involvement in local and remote myocardial ischemic conditioning. Shock.

[30] Xenocostas A, Hu H, Chin-Yee N, Lu X, Chin-Yee I, Feng Q (2010). Erythropoietin is equally effective as fresh-blood transfusion at reducing infarct size in anemic rats. Crit. Care Med..

[31] Hu H, Xenocostas A, Chin-Yee N, Lu X, Chin-Yee I, Feng Q (2012). Transfusion of fresh but not old stored blood reduces infarct size and improves cardiac function after acute myocardial infarction in anemic rats*. Crit. Care Med..

[32] Galluzzi L, Aaronson SA, Abrams J, Alnemri ES, Andrews DW, Baehrecke EH (2009). Guidelines for the use and interpretation of assays for monitoring cell death in higher eukaryotes. Cell Death Differ..

[33] Malhi H, Kaufman RJ (2011). Endoplasmic reticulum stress in liver disease. J. Hepatol..

[34] Groenendyk J, Sreenivasaiah PK, de Kim H, Agellon LB, Michalak M (2010). Biology of endoplasmic reticulum stress in the heart. Circ. Res..

[35] Zhang J, Wei C, Wang H, Tang S, Jia Z, Wang L (2013). Protective effect of qiliqiangxin capsule on energy metabolism and myocardial mitochondria in pressure overload heart failure rats. Evid. Based Complement. Altern. Med..

[36] Zhuo XZ, Wu Y, Ni YJ, Liu JH, Gong M, Wang XH (2013). Isoproterenol instigates cardiomyocyte apoptosis and heart failure via AMPK inactivation-mediated endoplasmic reticulum stress. Apoptosis.

[37] Terai K, Hiramoto Y, Masaki M, Sugiyama S, Kuroda T, Hori M (2005). AMP-activated protein kinase protects cardiomyocytes against hypoxic injury through attenuation of endoplasmic reticulum stress. Mol. Cell. Biol..

[38] Logue SE, Cleary P, Saveljeva S, Samali A (2013). New directions in ER stress-induced cell death. Apoptosis.

[39] Minamino T, Komuro I, Kitakaze M (2010). Endoplasmic reticulum stress as a therapeutic target in cardiovascular disease. Circ. Res..

[40] Zou XJ, Yang L, Yao SL (2012). Endoplasmic reticulum stress and C/EBP homologous protein-induced Bax translocation are involved in angiotensin II-induced apoptosis in cultured neonatal rat cardiomyocytes. Exp. Biol. Med..

[41] Wu XD, Zhang ZY, Sun S, Li YZ, Wang XR, Zhu XQ (2013). Hypoxic preconditioning protects microvascular endothelial cells against hypoxia/reoxygenation injury by attenuating endoplasmic reticulum stress. Apoptosis.

[42] Gan R, Hu G, Zhao Y, Li H, Jin Z, Ren H (2012). Post-conditioning protecting rat cardiomyocytes from apoptosis via attenuating calcium-sensing receptor-induced endo(sarco)plasmic reticulum stress. Mol. Cell. Biochem..

[43] Shintani Y, Kapoor A, Kaneko M, Smolenski RT, D'Acquisto F, Coppen SR (2013). TLR9 mediates cellular protection by modulating energy metabolism in cardiomyocytes and neurons. Proc. Natl. Acad. Sci. USA.

[44] Sasaki H, Asanuma H, Fujita M, Takahama H, Wakeno M, Ito S (2009). Metformin prevents progression of heart failure in dogs: Role of AMP-activated protein kinase. Circulation.

[45] Drewlo S, Johnson E, Kilburn BA, Kadam L, Armistead B, Kohan-Ghadr HR (2020). Irisin induces trophoblast differentiation via AMPK activation in the human placenta. J. Cell. Physiol..

[46] Li RL, Wu SS, Wu Y, Wang XX, Chen HY, Xin JJ (2018). Irisin alleviates pressure overload-induced cardiac hypertrophy by inducing protective autophagy via mTOR-independent activation of the AMPK-ULK1 pathway. J. Mol. Cell. Cardiol..

[47] Xin C, Zhang Z, Gao G, Ding L, Yang C, Wang C (2020). Irisin attenuates myocardial ischemia/reperfusion injury and improves mitochondrial function through AMPK pathway in diabetic mice. Front. Pharmacol..

[48] Fan J, Zhu Q, Wu Z, Ding J, Qin S, Liu H (2020). Protective effects of irisin on hypoxia-reoxygenation injury in hyperglycemia-treated cardiomyocytes: Role of AMPK pathway and mitochondrial protection. J. Cell. Physiol..

[49] Huh JY, Mougios V, Kabasakalis A, Fatouros I, Siopi A, Douroudos II (2014). Exercise-induced irisin secretion is independent of age or fitness level and increased irisin may directly modulate muscle metabolism through AMPK activation. J. Clin. Endocrinol. Metab..

[50] Chen K, Xu Z, Liu Y, Wang Z, Li Y, Xu X (2017). Irisin protects mitochondria function during pulmonary ischemia/reperfusion injury. Sci. Transl. Med..

[51] Lee HJ, Lee JO, Kim N, Kim JK, Kim HI, Lee YW (2015). Irisin, a novel myokine, regulates glucose uptake in skeletal muscle cells via AMPK. Mol. Endocrinol..

[52] Aydin S, Kuloglu T, Aydin S, Eren MN, Celik A, Yilmaz M (2014). Cardiac, skeletal muscle and serum irisin responses to with or without water exercise in young and old male rats: Cardiac muscle produces more irisin than skeletal muscle. Peptides.

[53] Xie C, Zhang Y, Tran TD, Wang H, Li S, George EV (2015). Irisin controls growth, intracellular Ca^2+^ signals, and mitochondrial thermogenesis in cardiomyoblasts. PLoS ONE.

